# Green tea extract and black tea extract differentially influence cecal levels of short‐chain fatty acids in rats

**DOI:** 10.1002/fsn3.607

**Published:** 2018-03-01

**Authors:** Tomonori Unno, Naomi Osakabe

**Affiliations:** ^1^ Department of Health and Nutrition Tokyo Kasei Gakuin University Chiyoda‐ku Tokyo Japan; ^2^ Department of Bio‐Science and Engineering Shibaura Institute of Technology Minuma‐ku Saitama‐shi Saitama Japan

**Keywords:** black tea, green tea, gut microbiota, short‐chain fatty acids, starch digestion

## Abstract

Increasing evidence indicates that gut microbiota plays a critical role to maintain the host's health. The biological function of microbially produced short‐chain fatty acids (SCFA) becomes the focus of attention. This study aimed to compare the effects of green tea extract (GTE) and black tea extract (BTE) on cecal levels of SCFA in rats. Rats consumed an assigned diet of either a control diet, a GTE diet (10 g/kg), or a BTE diet (10 g/kg), for 3 weeks. The dietary addition of GTE significantly reduced the concentrations of acetate and butyrate in cecal digesta compared to the control, but BTE showed an increased trend for a cecal pool. In the GTE group, a significant amount of undigested starch was excreted in feces, but BTE produced no effect. Interestingly, feces of rats fed the BTE diet contained higher bacterial 16S rRNA gene copy numbers for total eubacteria compared to the control diet. Taken together, treatments of the diets with GTE and BTE brought about a different degree of producing SCFA in rat cecum. BTE might advantageously stimulate more SCFA production than GTE by facilitating bacterial utilization of starch.

## 1 INTRODUCTION

More than 100 trillion bacteria live symbiotically with our intestine, building the collective community. Organisms dominating gut microbiota are members of the phyla Firmicutes and Bacteroidetes, the relative abundance of which varies among individuals (Eckburg et al., 2005). Attention is currently focused on the intricate role of intestinal microbial ecosystems in maturating immune system and modulating energy metabolism (Cénit, Matzaraki, Tigchelaar, & Zhernakova, 2014). It is recognized that environmental factors such as mode of birth, infant feeding patterns, antibiotic usage, and long‐term dietary habit influence the overall microbial community (Power, O'Toole, Stanton, Ross, & Fitzgerald, 2014). Diet clearly has a major impact on variation in the composition of gut microbiota. Prebiotics (e.g., indigestible oligosaccharides and dietary fibers) are resistant to enzymatic digestion and are fermented by the intestinal bacteria to generate short‐chain fatty acids (SCFA), consequently modulating selectively the growth and/or activity of intestinal bacteria linked to a variety of health benefits (Brownawell et al., 2012). SCFA themselves perform multiple functions not only as a fuel for colonic epithelial cells, but also as specific signaling molecules to regulate the host's metabolism (Byrne, Chambers, Morrison, & Frost, 2015). SCFA bind G protein‐coupled receptors, namely GPR41 and GPR43, which are expressed in enteroendocrine cells in the intestinal epithelium, and activate the secretion of peptide YY (PYY) and glucagon‐like peptide‐1 (GLP‐1), which are responsible for reducing food intake and increasing energy expenditure (den Besten et al., 2013). Over the past decade, research seeks to identify the roles of food constituents dynamically modulating the gut microbiota to produce SCFA.

Tea is one of the most consumed beverages worldwide. People in Japan and China preferably consume a hot infusion of green tea, whereas in most other countries they predominantly consume black tea. Both types of tea are produced from the leaves of the plant *Camellia sinensis*, but undergo different manufacturing processes. To produce green tea, freshly harvested leaves are rapidly steamed or pan‐fried to inactivate polyphenol oxidase. The production of black teas involves the enzymatic oxidation process of fresh leaves by withering. Green tea retains the original form of flavan‐3‐ols, consisting of epicatechin (EC), epigallocatechin (EGC), epicatechin‐3‐gallate (ECG), and epigallocatechin‐3‐gallate (EGCG), whereas in the manufacturing process of black tea, parts of the original flavan‐3‐ols convert to theaflavins and thearubigins (Graham, 1992). Only small parts of ingested flavan‐3‐ols and theaflavins enter the bloodstream, but greater parts of them pass to the large intestine where the bacteria convert them to ring‐fission catabolites (Clifford, van der Hooft, & Crozier, 2013).

Taking into account the growing evidence about the beneficial effects of tea polyphenols, despite the low bioavailability of these molecules, it is reasonable to consider that the nature of tea polyphenols–microbiota interaction may govern the host's health. Based on a newly emerging concept, a few published review articles have shown the interactive potential of tea polyphenols (effect of gut microbiota on the catabolism of tea polyphenols and the effect of tea polyphenols on the modulation of gut microbiota) (van Duynhoven et al., 2013; Etxeberria et al., 2013). The modulations of gut microbiota by tea polyphenols may be potentially relevant to SCFA formation in the large intestine. A previous study by our group demonstrated that the dietary addition of EGCG (6 g/kg), the most predominant polyphenol in green tea, lowered cecal levels of acetate and butyrate in rats (Unno, Sakuma, & Mitsuhashi, 2014). Recent investigation also revealed that the addition of green tea extract (GTE) (2 g/L) to drinking water increased wet mass of cecal digesta and decreased cecal SCFA concentrations in mice (Ward et al., 2017). Interestingly, regarding black tea extract (BTE), an *in vitro* model reflective of the distal region of the human large intestine recorded a different result suggesting that a continuous dose BTE may be capable of significantly increasing the levels of acetate during the fermentation (van Dorsten et al., 2012). However, there have been few *in vivo* comparison trials on the composition and activity of gut microbiota between GTE and BTE. Therefore, this study was intended to compare the effects of GTE and BTE on the compositions of fecal microbiota and the cecal levels of SCFA in rats.

## 2 MATERIALS AND METHODS

### 2.1 Preparations of tea extracts

Japanese green tea (Yabukita variety) and Indian black tea (Assam variety) were purchased commercially from the Japan market. They were extracted with 20 volumes of aqueous ethanol (395 g/l) overnight at room temperature. After filtration, the extraction liquid was evaporated under reduced pressure and then freeze‐dried. As dietary caffeine caused a significant decrease in food intake at the beginning of feeding (Naismith, Akinyanju, & Yudkin, 1969), we further prepared decaffeinated tea extracts. To remove caffeine, an aliquot of the dried samples was again dissolved in water and extracted with 20 volumes of chloroform twice. The resultant aqueous phase was evaporated and then freeze‐dried to obtain a decaffeinated GTE and a decaffeinated BTE.

### 2.2 Determinations of total polyphenols, flavan‐3‐ols, and theaflavins

Total polyphenol contents of GTE and BTE were determined by the iron tartrate colorimetric method (Iwasa, 1975) and the Folin–Ciocalteu method (Singleton & Rossi, 1965), respectively. The contents of flavan‐3‐ols, caffeine, and theaflavins were measured by a high‐performance liquid chromatography (HPLC) system. Flavan‐3‐ols and caffeine were separated on an InertSustain C18 analytical column (4.6 mm ID × 150 mm length, 3 μm particle size) from GL Sciences Inc. (Tokyo, Japan), with a temperature at 40°C. The mobile phase solvent was composed of water (850 ml), acetonitrile (150 ml), and phosphoric acid (1 ml). Elution was performed with a flow rate of 1.0 ml/min, and absorbance wavelength at 230 nm was monitored throughout each run. Theaflavins were separated by a gradient‐elution technique of using two solvents: (A) a mixture of water (950 ml), acetonitrile (50 ml), and phosphoric acid (0.5 ml) and (B) a mixture of water (500 ml), acetonitrile (500 ml), and phosphoric acid (0.5 ml). The mobile phase composition started at 100% solvent A, being increased linearly to 30% solvent B in 20 min, maintained 30% solvent B for 15 min, and followed by a linear increase of solvent B to 100% in 25 min. A two‐solvent gradient elution was performed with a flow rate of 1.0 ml/min, and absorbance wavelength at 375 nm was monitored. Standard reagents of EC, EGC, ECG, EGCG, and theaflavin (TF), theaflavin‐3‐gallate (TF3G), theaflavin‐3′‐gallate (TF3′G), theaflavin‐3,3′‐digallate (TFdiG) were purchased from Wako Pure Chemical Industries, Ltd. (Osaka, Japan), and caffeine from Nakalai Tesque, Inc. (Kyoto, Japan).

### 2.3 Animals and diets

Four‐week‐old, male Wistar rats (*n* = 21) were purchased from Tokyo Laboratory Animals Science Co., Ltd. (Tokyo, Japan) and were acclimated for 1 week in stainless steel cages at 22°C in a room with an automatically controlled 12‐hr lighting cycle. During the acclimation period, the rats were fed a commercial chow (type MF, Oriental Yeast Co., Ltd., Tokyo, Japan). They were divided into three groups (*n *= 7 per group) and were fed their respective diet: a control diet, a GTE diet, or a BTE diet (Table 1). They were given free access to the experimental diets and tap water for 3 weeks. Feces were collected weekly. At the final day of the experiment, rats were humanely killed by inhaling high levels of carbon dioxide, and the cecum was immediately excised. The cecal digesta was stored at −40°C until use.

**Table 1:** Diet composition (g/kg)

	Experimental diet		
	Control	GTE	BTE
Casein	200	200	200
dl‐Methionine	3	3	3
Corn starch	550	550	550
Sucrose	100	100	100
Cellulose powder	50	40	40
Corn oil	50	50	50
Mineral mix (AIN‐76)	35	35	35
Vitamin mix (AIN‐76)	10	10	10
Choline bitartrate	2	2	2
Decaffeinated green tea extract (GTE)	—	10	—
Decaffeinated black tea extract (BTE)	—	—	10

### 2.4 SCFA concentrations in cecal digesta

An aliquot of cecal digesta was mixed with 4 volumes of distilled water to prepare the homogenate. Acetate, propionate, and butyrate in the homogenate were derivatized with 2‐nitrophenylhydrazine hydrochloride in the presence of 1‐ethyl‐3‐(3‐(dimethylamino)propyl)carbodiimide hydrochloride and were measured by the HPLC system (Miwa, Hiyama, & Yamamoto, 1985). The derivatives were separated on an YMC‐Pack type FA column (6.0 mm ID × 250 mm length) from YMC Co., Ltd. (Kyoto, Japan) and were detected with a variable wavelength monitor with the absorbance set at 400 nm. The mobile phase was composed of water (540 ml), acetonitrile (300 ml), and methanol (160 ml) with a flow rate of 1.2 ml/min at 50°C.

### 2.5 Fecal bacterial 16S rRNA gene copy number

Fecal samples collected during the third week of feeding were ground by a grinder mill (type TML161, Tescom Co., Ltd., Tokyo, Japan). DNA was extracted according to the method of Takahashi, Tomita, Nishioka, Hisada, and Nishijima (2014). The quantification of 16S rRNA gene copies for total eubacteria was performed by a quantitative real‐time PCR using 341F (5′‐CCTACGGGAGGCAGCAG‐3′) and 534R (5′‐ATTACCGCGGCTGCTGG‐3′) primers (Muyzer, de Waal, & Uitterlinden, 1993). Amplification reactions were performed by a Rotor‐Gene Q apparatus (Qiagen, Germany) using the SYBR Premix Ex TaqII (Tli RNaseH Plus, Takara Bio, Shiga, Japan). The copy number was calculated from the standard curve that was generated from standard bacterium *Escherichia coli* JCM1649^T^.

### 2.6 Fecal bacterial composition

Fecal samples of rats were analyzed by targeting the bacterial 16S rRNA genes using a terminal restriction fragment length polymorphism (T‐RFLP) technique according to the procedure described by Nagashima, Mochizuki, Hisada, Suzuki, and Shimomura (2006). The 6‐FAM fluorescently labeled primers 516F (5′‐TGCCAGCAGCCGCGGTA‐3′) and 1510R (5′‐GGTTACCTTGTTACGACTT‐3′) were used to amplify the 16S rRNA genes. The purified PCR products were digested with the restriction enzyme Bs*l*I (New England BioLabs Japan, Inc., Tokyo, Japan). The length of the terminal restriction fragments was determined using an automated sequence analyzer (ABI PRISM 3130xl DNA Sequencer, Applied Biosystems, Carlsbad, CA, USA). The abundance of each terminal restriction fragment was determined on the basis of fluorescence intensity using the software GeneMapper ver. 4 (Applied Biosystems). Fragment sizes were assigned to categories of operational taxonomic units (OTU). The OTU data were used to identify the phylotypes by matching to those predicted from various phylotypes in the literature (Nagashima et al., 2006).

### 2.7 Fecal concentrations of starch

The concentrations of undigested starch in feces were determined after hydrolysis with thermostable α‐amylase and amyloglucosidase, followed by an enzymatic colorimetric measurement of the released glucose using a commercial assay kit (Glucose CII‐test, Wako Pure Chemical Industries, Ltd.) (Unno, Hisada, & Takahashi, 2015). Results were converted to a starch basis by multiplying them by 0.9.

### 2.8 Inhibition of pancreatic α‐amylase in vitro

The reaction mixture consisted of 0.2 ml of tea extracts at different concentrations (range from 0.1 to 50 g/l), 0.7 ml of starch solution (10 g/l in 25 mM sodium phosphate buffer containing 6 mM sodium chloride at pH 6.9), and 0.1 ml of porcine pancreatic extract solution (1 g/l in same buffer as starch solution), and was incubated for 30 min at 37°C. Soluble starch and porcine pancreatic extract were purchased from Wako Pure Chemical Industries, Ltd. The reaction was then stopped with 0.2 ml of 100 g/l trichloroacetic acid. After centrifugation, the supernatant was passed through disposable solid‐phase extraction column cartridges (Strata‐X, 30 mg/ml, Phenomenex, Inc., Torrance, CA, USA), and 0.4 ml of the passed eluate was added to the same volume of acetonitrile. After centrifugation at 5,000 *g* for 10 min, liberated maltose in the supernatant was measured by the HPLC system (Unno et al., 2015). The rate of enzyme inhibition (%) was calculated as maltose concentration in the resulting mixture relative to the control (without GTE or BTE).

### 2.9 Statistical analysis

All data were represented as mean values and standard deviations. Significant differences among the groups were determined by one‐way ANOVA, followed by Tukey's test. The bacterial 16S rRNA gene copy numbers were converted into logarithm before statistical analysis. For evaluation of the difference in percentage composition of fecal microbiota among groups, the Kruskal–Wallis test, followed by Dunn's multiple comparison test, was adopted. These statistical analyses were performed by a commercial software package (GraphPad Prism 5 for Windows, GraphPad Software, Inc., San Diego, CA, USA). Differences were considered significant at *p *<* *.05.

## 3 RESULTS

### 3.1 Polyphenol content in tea extracts

Extraction of the commercial teas with aqueous ethanol and subsequent caffeine removal process yielded 25.0% (w/w) for GTE and 26.3% (w/w) for BTE from the original leafy tea materials. The contents of total polyphenols, flavan‐3‐ols, and theaflavins in the respective samples were shown in Table 2. GTE showed slightly higher total polyphenol value than BTE, but each flavan‐3‐ol component differed vastly between the two samples. The sum of EC, EGC, ECG, and EGCG in GTE was 275 g/kg for GTE and 35 g/kg for BTE. EGC was present in the highest concentration in GTE, followed by EGCG. In BTE, total theaflavin (the sum of TF, TF3G, TF3′G, TFdiG) was 35 g/kg. Other polyphenolic constituents in BTE may be attributed to thearubigins. In this study, however, thearubigins were chromatographically irresolvable. Caffeine concentrations dropped to less than 10 g/kg.

**Table 2:** Contents of total polyphenols, flavan‐3‐ols, theaflavins, and caffeine in tea extracts (g/kg)

	Tea extract	
	GTE	BTE
Total polyphenols	417	374
Flavan‐3‐ols		
Epicatechin (EC)	28	4
Epigallocatechin (EGC)	123	3
Epicatechin‐3‐gallate (ECG)	25	15
Epigallocatechin‐3‐gallate (EGCG)	99	13
Theaflavins		
Theaflavin (TF)	2	5
Theaflavin‐3‐gallate (TF3G)	0	11
Theaflavin‐3′‐gallate (TF3′G)	0	6
Theaflavin‐3,3′‐digallate (TFdiG)	0	13
Caffeine	5	9

### 3.2 Body weight gain and cecal digesta mass

The addition of GTE or BTE at the concentrations of 10 g/kg in the diet had little influence on food intake throughout the experimental periods (Table 3). Although there were no significant differences in body weight gain among the groups, both dietary treatments led to significant increases in wet mass of cecal digesta per 100 g of body weight compared to the control (*p *<* *.01 for GTE, *p *<* *.05 for BTE). The relative mass of cecal digesta for the GTE group also statistically differed from that for the BTE group (*p *<* *.05).

**Table 3:** Body weight gain, food intake, and wet mass of cecal digesta of rats fed the experimental diet for 3 weeks

	Control	GTE	BTE
Initial body weight (g)	157 ± 6	158 ± 6	157 ± 8
Final body weight (g)	329 ± 14	318 ± 32	318 ± 21
Body weight gain (g)	172 ± 13	160 ± 27	161 ± 18
Food intake (g)	505 ± 22	483 ± 42	490 ± 35
Cecal digesta mass (g/kg bw)	7.5 ± 1.5 c	12.1 ± 1.0 a	9.7 ± 2.1 b

Values are the mean ± standard deviations (*n *=* *7). Treatments with different letters are significantly different (*p *<* *.05).

### 3.3 SCFA concentration in cecal digesta

**Figure 1:**
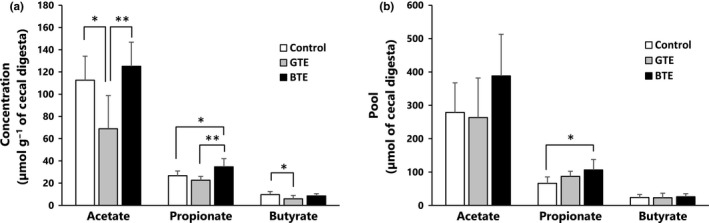
Effect of GTE and BTE on the concentration (a) and the pool (b) of SCFA in cecal digesta of rats. Rats were fed for 3 weeks with either a control diet, a GTE (10 g/kg) diet, or a BTE (10 g/kg) diet. The cecal SCFA pool (μmol) = SCFA concentration (μmol/g) × cecal digesta (g). Values are means with standard deviations represented by vertical bars (*n *= 7). Asterisks indicate significant difference: *p <* *.05, **p <* *.01

The addition of GTE in the diet resulted in significant decreases in the cecal concentrations (μmol/g of cecal digesta) of acetate and butyrate compared to the control (*p *<* *.05 for both) (Figure 1a). BTE turned out entirely different from GTE. There were statistically significant differences in the cecal concentrations of acetate and propionate between GTE and BTE (*p *<* *.01 for both). BTE also produced an increase in propionate compared to the control. Such statistical significances by GTE disappeared when expressed as μmol per whole cecal digesta (Figure 1b).The SCFA pool (μmol) was computed by multiplying the SCFA concentration (μmol/g of cecal digesta) by the mass (g) of cecal digesta collected. Dietary BTE resulted in the increase only in propionate compared to the control group (*p *<* *.05).

### 3.4 Modulation of fecal microbiota

**Figure 2:**
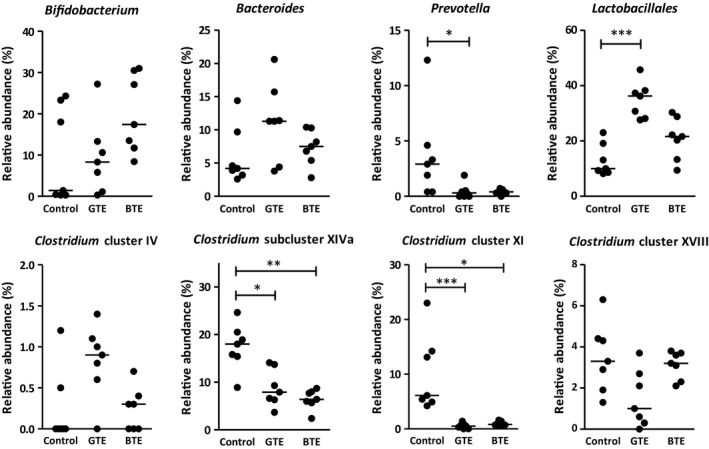
Effect of GTE and BTE on the profile of fecal microbiota. Feces of rats fed either a control diet, a GTE (10 g/kg) diet, or a BTE (10 g/kg) diet were collected during the third week of feeding. Values are expressed as percentages of the peak area of a particular OTU to the total peak area of all OTU. Horizontal bars indicate median (*n *=* *7). Asterisks indicate statistical significance: *p <* *.05, **p <* *.01, ***p <* *.001

Treatments resulted in a slight rise in dry mass of feces collected during the third week of feeding, but there were no statistical significance among the groups (Table 4). Table 4 also shows logarithmically transformed bacterial 16S rRNA gene copy number per gram of feces collected during the third week of feeding, with the BTE exhibiting a significantly higher level than the control group (*p *<* *.05). When converted into the copy number per 7‐day feces, the BTE group showed significant differences from the control group (*p *<* *.01) and the GTE group (*p *<* *.05). Figure 2 displays the profiles of fecal microbiota composition, indicating that both treatments of the diet with tea extracts made major impacts on the taxonomic composition of the fecal microbiota. The GTE diet significantly reduced the relative abundance of *Prevotella* (*p *<* *.05), *Clostridium* subcluster XIVa (*p *<* *.05), and *Clostridium* cluster XI (*p *<* *.001), but increased *Lactobacillales* (*p *<* *.001) compared to the control diet. The BTE diet also showed significant reductions in *Clostridium* subcluster XIVa (*p *<* *.01) and *Clostridium* cluster XI (*p *<* *.05). Both treatments slightly raised median values of the *Bifidobacterium* abundance, although they did not reach statistical significance.

**Table 4:** Bacterial log _10_ 16S rRNA gene copy numbers and starch excretion in feces of rats fed the experimental diets

	Control	GTE	BTE
Dried feces mass (g/wk)	13.2 ± 1.0	14.8 ± 2.2	15.1 ± 1.4
Log _10_ 16S rRNA gene copy number			
per g dry feces	13.44 ± 0.12 b	13.47 ± 0.13 ab	13.60 ± 0.06 a
per weekly dry feces	14.56 ± 0.09 b	14.63 ± 0.12 b	14.77 ± 0.07 a
Fecal starch excretion (g/wk)	0.16 ± 0.04 b	0.42 ± 0.18 a	0.14 ± 0.03 b

Feces were collected during the third week of feeding. Values are the mean ± standard deviations (*n *=* *7). Treatments with different letters are significantly different (*p* < .05).

### 3.5 Fecal outputs of starch

Compared to the control diet, the GTE diet brought about a significant increase in starch excretion in the feces (*p *<* *.001), whereas the BTE diet exhibited no effect. The feces of rat which consumed the GTE contained more starch compared to the BTE (*p *<* *.001).

### 3.6 Inhibition of α‐amylase

**Figure 3:**
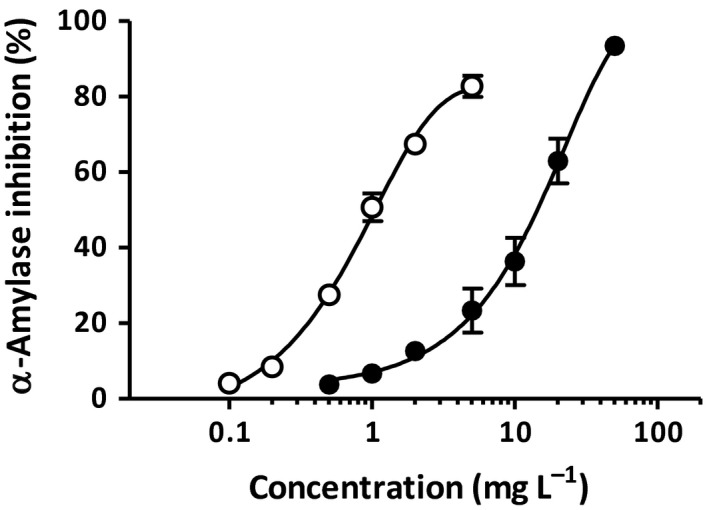
Dose‐dependent inhibition of GTE and BTE against pancreatic α‐amylase activity. Values are means with standard deviations represented by vertical bars (*n *=* *3). Solid circles and open circles indicate GTE and BTE, respectively

Green tea extract and BTE inhibited the enzyme activity of porcine pancreatic α‐amylase in a concentration‐dependent manner (Figure 3), with the effect of BTE being stronger than GTE.

## 4 DISCUSSION

Growing evidence leads us to understand that the composition and activity of gut microbiota may be relevant to health and diseases. Several strategies, including the intake of polyphenol‐rich foods (Queipo‐Ortuño et al., 2012; Tzounis et al., 2011), have been proposed to modulate the gut microbiota in humans. Tea is also an excellent source of polyphenols, but types of polyphenol differ substantially among types of tea. Green tea predominantly contains flavan‐3‐ols, and black tea their polymerized ones. Parts of consumed tea polyphenols reach the large intestine where they interact with colonic bacteria (Hamdaoui et al., 2016), but information regarding their impacts on the composition and activity of gut microbiota is limited.

Rodent model studies demonstrated biological effects with ingested doses of GTE or EGCG ranging from 0.1 to 25 g/kg of the diet (Crespy & Williamson, 2016). The present study employed the respective tea extracts at the mass composition of 10 g/kg of the diet. Although food intake was comparable among the groups, the addition of GTE or BTE to the diet brought about a common distinctive feature of increasing wet mass of cecal digesta compared to the control group. However, only the GTE diet led to a major decreasing action in SCFA concentrations in cecal digesta on a wet mass basis. Compared to the control group, the GTE group showed significant decreases in the cecal concentrations of acetate and butyrate, although their cecal pools returned to the control level. The same results were seen in previous studies in rats fed a diet supplemented with 6 g/kg EGCG (Unno et al., 2014) and in mice given a drinking water supplemented with 2 g/l GTE (Ward et al., 2017). It is known that acetate is produced by a variety of gut bacteria, but butyrate and propionate are produced by a distinct subset of bacteria. The predominant butyrate producers belong to the Firmicutes phylum, particularly members of *Clostridium* cluster IV, and XIVa, while propionate is produced by Bacteroidetes phylum (Louis & Flint, 2017). In the present study, the T‐RFLP analysis revealed that treatment of diet with 10 g/kg GTE significantly modulated fecal microbiota, as typified by decreases in the relative abundance of *Clostridium* subcluster XIVa and *Clostridium* cluster XI, but had little impact on *Bacteroides*. Provided that GTE has the potential to modulate the balance of gut microbiota selectively, it may possibly be that the decreased abundance of *Clostridium* subcluster XIVa resulted in the reduction in cecal butyrate concentration. Because GTE had an insignificant effect on propionate producer *Bacteroides*, cecal propionate concentration remained nearly unchanged.

Despite significant decreases in the relative abundance of *Clostridium* subcluster XIVa and *Clostridium* cluster XI, the BTE group showed an increasing trend in the cecal pool of SCFA rather than a decreasing one. One possibility is that more undigested dietary starch could have reached the large intestine where bacteria may be fermentatively utilized to produce SCFA. The result of *in vitro* experiment with α‐amylase inhibition supports such a hypothesis. BTE had an inhibitory effect on pancreatic α‐amylase. In particular given that BTE could limit intestinal starch digestion via α‐amylase inhibition under *in vivo* condition, with its effect being stronger than GTE, it may be safely assumed that BTE has a facilitating role in transferring undigested starch into the large intestine. In addition, the fecal starch level also helps account for the possibility that the degree of microbial starch utilization might differ among the experimental groups. The feces of rats which consumed the GTE diet contained a significant amount of starch, whereas that of BTE group had a smaller amount. This distinct difference in fecal starch content may also reflect the fact that intestinal bacteria in the BTE group intensively utilize undigested dietary starch as a substrate for producing SCFA. Another point to notice is a major significance in fecal copy number of target gene. Real‐time PCR analysis revealed that total 16S rRNA gene copy numbers were significantly higher in the BTE group than in the control group. This implies that treatment of the diet with BTE may enhance the absolute microbial count of eubacteria in feces, even though the relative abundance of *Clostridium* subcluster XIVa was decreased. Presumably, undigested starch might serve as an additional bacterial growth substrate. For detailed reasons that are unknown, this finding may well be related in part to the increased trend of SCFA pools in cecal digesta of the BTE group. It is here to be noted that tea polyphenols possess antimicrobial effects against a certain type of organism by binding to the bacterial lipid bilayer cell membrane, thereby disturbing membrane function, and consequently inhibiting cell growth (Reygaert, 2014). An *in vitro* experiment also showed that both GTE and BTE have an antimicrobial effect, with BTE being weaker than GTE (Chou, Lin, & Chung, 1999). From the results of this *in vivo* study, it is possible to understand that the dietary addition of BTE (10 g/kg) exerted little antimicrobial effect against intestinal bacteria.

Only a few trials to date have demonstrated the clinical impact of dietary tea polyphenols on the gut microbiota, but the results have been inconsistent. Okubo et al. (1992), for example, reported that administration of 0.4 g of green tea polyphenols 3 times a day resulted in reducing *Clostridium* perfringens and other *Clostridium* spp. during the intake periods, whereas percentages of *Bifidobacterium* spp. in total fecal counts markedly increased. They also found significant increases in fecal levels of acetate and propionate. Jin, Touyama, Hisada, and Benno (2012) also reported that 1000 ml of green tea drinking for 10 days caused an increasing trend in fecal *Bifidobacterium* spp. In a different study, a 12‐week ingestion of 1.80–1.97 g of green tea polyphenols along with 0.28–0.45 g of caffeine per day showed no significant difference in the composition of gut bacteria (Janssens et al., 2016). In the investigation of the effects of black tea drinking, Mai et al. (2004) showed that the bacterial number of *Clostridium* cluster XIVa group detected by a fluorescent in situ hybridization method was not affected. To evaluate in more detail the effects of GTE and BTE on the selective growth of the intestinal microbiota, as well as the fecal SCFA levels, further clinical studies under controlled conditions are needed on the amount of tea polyphenols daily consumed, the formulation of samples, initial microbial diversity, and the dietary habits of subjects.

## 5 CONCLUSION

The present study demonstrated that dietary administration of decaffeinated extracts of green tea and black tea to rats clearly affects cecal levels of SCFA in a different manner. Compared to the control, treatment of the diet with 10 g/kg GTE significantly decreased the concentrations of SCFA in cecal digesta, whereas 10 g/kg BTE functioned more like an enhanced trend. Underlying mechanisms may be proposed based on the microbial effects of GTE and BTE. Both tea extracts could alter the microbial composition selectively, whereas BTE enlarged the microbial counts. As to the BTE effect, a significant amount of undigested starch might contribute to the microbial growth and fermentatively with the production of SCFA.

## CONFLICT OF INTERESTS

The authors declare that they do not have any conflict of interest.

## ETHICAL REVIEW

The animal care and experimental procedures were approved by the Animal Research Committee of Tokyo Kasei Gakuin University (approval no. H28‐3) and conducted according to the guidelines of the Management of Laboratory Animals in Tokyo Kasei Gakuin University.
